# DNA Movies and Panspermia

**DOI:** 10.3390/life1010009

**Published:** 2011-10-20

**Authors:** Victor Norris, Yohann Grondin

**Affiliations:** 1 EA 3829, Department of Biology, University of Rouen, 76821 Mont Saint Aignan, France; 2 Harvard School of Public Health, 665 Huntington Avenue, 02115 Boston, MA, USA; E-Mail: ygrondin@hsph.harvard.edu

**Keywords:** origins of life, translation, hyperstructure, Pioneer, cholesteric phase

## Abstract

There are several ways that our species might try to send a message to another species separated from us by space and/or time. Synthetic biology might be used to write an epitaph to our species, or simply “Kilroy was here”, in the genome of a bacterium via the patterns of either (1) the codons to exploit Life's non-equilibrium character or (2) the bases themselves to exploit Life's quasi-equilibrium character. We suggest here how DNA movies might be designed using such patterns. We also suggest that a search for mechanisms to create and preserve such patterns might lead to a better understanding of modern cells. Finally, we argue that the cutting-edge microbiology and synthetic biology needed for the Kilroy project would put origin-of-life studies in the vanguard of research.

## 1. Introduction

The urge to leave traces of ourselves is revealed by the pictures in our museums, by the books in our libraries and by the tags on the walls in our cities. This urge led to the message written onto the gold-anodised aluminium plaques on the Pioneer 10 and 11 probes sent out by NASA. The same urge might be harnessed to send a message to future species on our own planet. The question would then arise as to how such a message might be written. Attempts to answer this question risk crossing the line that separates science from science fiction. Sometimes, however, breaching the divide between scientific speculation and science fiction can be desirable. Indeed, it has been encouraged by the physicist and science fiction writer, Gregory Benford, who proposed that there is “a link between the science I practise, and the fiction I deploy in order to think about the larger implications of my work, and of others'.” [1]. In other words, allowing one's imagination to explore new possibilities in the writing of science fiction can be of value to real science. We use this to license the following series of speculations about intelligent life in the universe, its likely desire to communicate, and the use we might make of synthetic biology to write “Kilroy was here” in the genome of a bacterium or other organism as an epitaph to *Homo sapiens*.

## 2. The Problem

It is conceivable that “intelligent”, dominant life-forms like ours have arisen previously on Earth. It is even conceivable that they have arisen—and will continue to arise—many times. The problem for species such as *Homo sapiens* (or, as we might prefer to call it, *Homo systemicus*) is that the selection for tribalism, aggression, power-seeking and, above all, obedience to the group (*i.e.* uncritical adoption of the group's beliefs and values), that leads to the species dominance is also likely to lead to the species destruction. It might be argued that no evident trace of such species has been found, as yet, in the fossil record. This might seem a powerful counter-argument given the effects of *Homo sapiens* on the ecosystem (e.g. via the relative abundance of pollens) or on fossilised artefacts (e.g. via our sophisticated tools). A possible explanation for this would be that such species have destroyed themselves so rapidly that they have left little trace behind. *Homo sapiens* may have lasted longer than most because its low intelligence relative to earlier species has retarded its development of weapons of mass destruction (e.g. of psychic, literally mind-blowing, weapons). Given awareness of its transience, an intelligent species (like many individuals) may want to want to leave a message for a future species, either just a “Kilroy was here” or some more interesting “message in a bottle”. But how could they do it so as to ensure that it could be read after tens or hundreds of millions of years?

## 3. Possible Approaches

One way would be to create artefacts on Earth along the lines of a modern equivalent of the pyramids. It is unlikely though that such artefacts could be constructed to last more than a few tens of thousands of years rather than the few hundreds of millions of years that be may needed for them to be interpreted [2]. The precursors of the pyramids, the mastabas, are already in a poor state despite the good conditions for preservation that have prevailed in Egypt. And little that we might construct is likely to survive a trip down a subduction zone. Another way would be to leave the message somewhere in the Solar System, perhaps to put it in a Lagrange Point, where one might hope it would stay for some time, or to send it off into the great black yonder as in the case of Pioneer 10 and 11. Yet another way would be to make use of biology.

## 4. The Biological Solution

**Figure 1:**
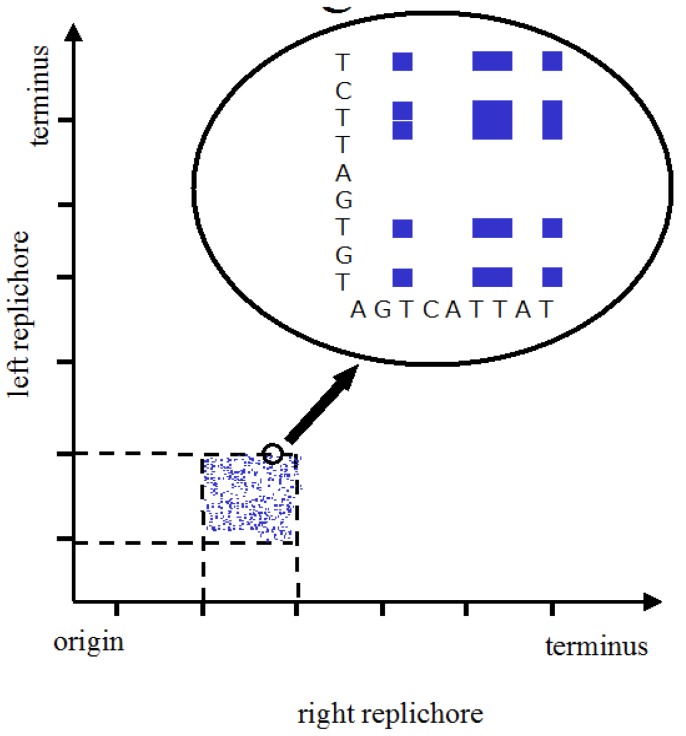
Matrix of thymine-thymine (T,T) pairs. The DNA sequences of the right and left replichores of *E. coli* constitute the axes. A zoom of a region is shown in which each (T,T) pair is coloured blue.

Bacteria have the advantage of being able to maintain themselves unscathed over millions of years in different conditions in which, to take the extremes, they can either grow by faithful reproduction or survive by sporulation. How then might bacteria be used to send a message across the aeons or the light years? There are several possibilities, including those involving fluorescent proteins [3], but one attractive possibility would be to write the message in the DNA of an organism that is likely to be sequenced. This raises the question of how to encode the message. It would be possible to create a pattern in the DNA to be detected by reading linearly but interpretation might prove difficult. A 2-D pattern would make patterns easier to both detect and interpret (just as a graph is a good way to analyse data). Suppose, for example, we were to take a circular bacterial chromosome like that of *Escherichia coli* and to use the sequences of the two replichores (*i.e.* the two *oriC-terC* sequences) as the axes of a 2-D matrix. (There are, of course, other possibilities such as taking the entire linear sequence from *oriC* back to *oriC* and then using that same sequence for both axes of the 2-D matrix). If one were to attribute a colour to each nucleotide base pair combination of x,y coordinates, this might be used to make a pretty pattern (Figure 1). The advantage of this 2-D visualisation method over a 1-D analysis can be grasped by considering that the folding that results in a particular pattern depends on the length of the line which in turn may depend on the particular proteins that bind to particular sequences; in this case, the 1-D analysis would have to tell us in an obvious way which protein is being expressed and what its binding specificities are. As far as we know, this not the case.

**Figure 2:**
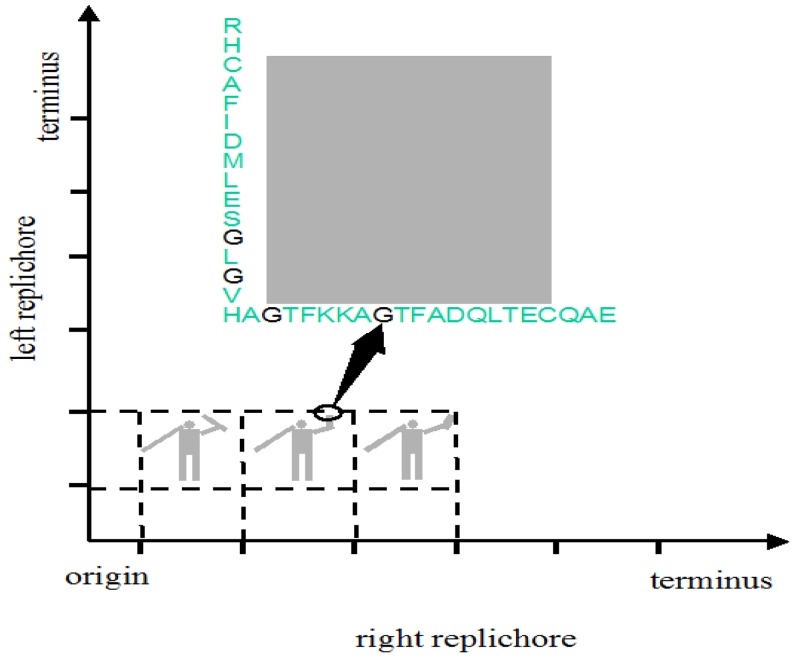
Successive frames showing a man waving. The right and left replichores have punctuation marks that can define successive frames. Within each frame, a grey block corresponds to the rectangle made by a sliding window along each axis in which a sequence of ten amino acids containing one or more glycines (G) is found in both the right and left replichores.

**Figure 3:**
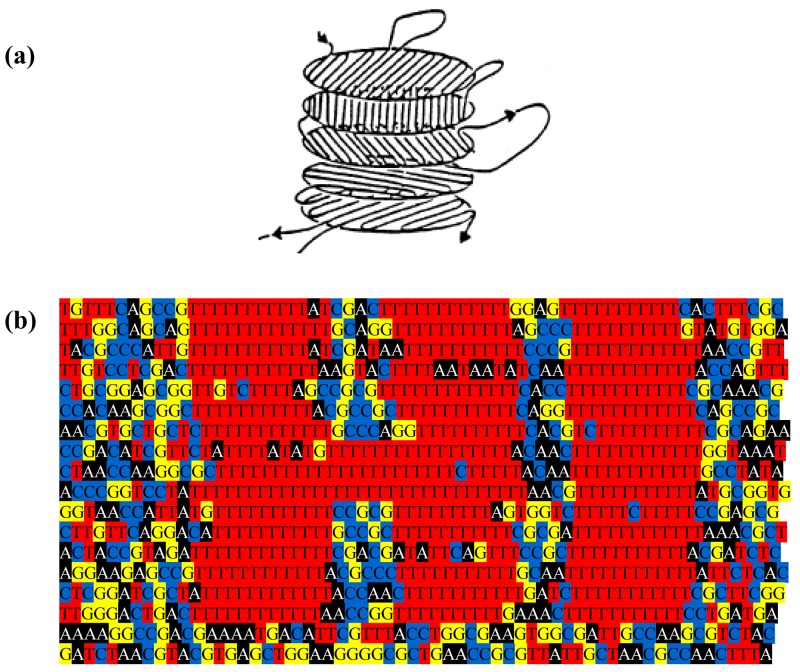
DNA sequence in a cholesteric or other ordered phase. (**a**) Successive planes of DNA in a cholesteric structure (taken from [4]) (**b**). The length of each line of sequence corresponds to the length that the sequence would take within a structure such as the bacterium or the head of the phage (where the length would equal the diameter, see text).

The genome might be used to make a still prettier pattern if one were to use pairs of amino acids as combinations or simply pairs of similar/identical amino acids. Obvious candidates include amino acids like cysteine that, via the disulphide bridge, are important in structuring proteins, or amino acids like glycine that, because they are common, might be used to colour the short windows in which they occur. It might make a more interesting and accessible code if use were made of natural punctuation marks in the chromosome. Such punctuation marks constitute a key to decryption that should be evident to species similar to ours (note that suspecting that a sequence contains a message is the essential step). Then the sequence could be divided up so as to make a series of frames and a movie could be made out of it (Figure 2). Finally, one might attribute a colour to each base and to arrange these bases in sequence in a series of lines in which the length of each line corresponds to some physical parameter such as the diameter of the chromosome, as determined perhaps by the diameter of the cell (Figure 3); if each line in Figure 3b represents a single filament in a cholesteric structure (Figure 3a, adapted from [4]) then the end of the line wraps round to continue on the line below (so successive lines would be read from left to right then right to left then right to left, *etc.*). In this figure, a block of thymines is coloured red but it might be judicious to choose the consensus sequence constituting a binding site on the DNA or the wide range of sequences that encode a common structure in a protein such as an α-helix, the prediction of which is an ongoing task [5].

## 5. Implementation of DNA Movies

“Punctuation marks” are the basis of the solenoid model for chromosome folding via the DNA-binding sites for sequence-specific transcriptional regulators which are located at regular distances that are multiples of 1/50th of the chromosome length [6]. Other periods such as the 33 kb in *E. coli* have been revealed by analysis of its ‘core’ genes and may be based on requirements for translation and possibly transcription [7]. These are not the only results (see for example [8,9]). Overall, periodicities may reflect two or more opposing constraints acting on the system. For example, there may be one constraint for expressed DNA and a different constraint for unexpressed DNA.

The first, non-equilibrium type, constraint might correspond to maximising the efficiency of translation by, for example, having all the codons for a particular amino acid translated near one another, which might be achieved by a particular set of 3-D distributions of codons within the cytoplasm and hence a particular set of 1-D distributions of these codons along the chromosome.

The second, equilibrium-type, constraint might correspond to optimising a reversible packing of DNA that would be obtained by the spontaneous adoption of a cholesteric structure as guided perhaps by sequences favouring high curvature [10] at the end of the loops or by other, as yet known, factors [11,12,13]. Homologous pairing of two double-stranded DNA molecules occurs *in vitro* and it has been proposed that direct recognition of homology between chemically intact B-DNA molecules should be possible *in vivo* [12,13]. Possible explanations—and there are many [14,15] —must be related in some way to the primary nucleotide sequence and might include, for example, sequence-dependent base-flipping [16,17] and braiding, which refers to any winding of two DNA molecules around each other driven by chiral electrostatic interactions and which has been invoked to explain homologous pairing [15]. Recent pairing experiments with DNA containing homologous and non-homologous regions are consistent with a sequence-dependent, long-range interaction and a sequence-independent, short-range interaction [18]. Such mechanisms might help keep regions of the genome together when they are not being expressed because it is advantageous for them to be together when they are expressed. The point here is that there are mechanisms that might be used by cells to produce ordered DNA structures even in quasi-equilibrium conditions. It would be interesting to learn whether a periodicity due to individual nucleotides and their complements is particularly evident in DNA that is largely untranscribed as in much of the DNA found in some dinoflagellates [19,20].

Irrespective of the exact nature and function of natural periodicities, the idea here is to exploit them as the frames in which a message is encoded. In one type of coding, this message would be in the position of pairs of amino acids in the two *ori-ter* arms or replichores. This would require extensive modification of existing coding sequences. It would require genes to be shifted from one location to another and for the sequences of individual genes to be extensively modified. But it is feasible. Even though the constraints on the choice of amino acids to form protein structures are strong (probably stronger than on the choice of nucleotides to form DNA structures) there is some evidence that protein structures may be easier to obtain than once believed [21]. Even without changing the nature of the amino acid, codon optimisation has led to improved translation rates, protein yields, and enzymatic activities (for references see [22]). For example, the genes encoding nitrogen fixation in *Klebsiella* have been extensively modified with codons being exchanged and unwanted sites for regulation being removed [23].

## 6. Difficulties to be Overcome

One difficulty is that the synthetic messenger, which we might term *Escherichia nuntius* or *Nostoc nuntius* (depending on its origin), is likely to undergo so many recombinations, rearrangements and mutations that the message will be lost. A partial solution might be to use a slimmer version of *E. coli* from which elements that favour recombination—including insertion sequence elements, transposases, defective phages, integrases, and site-specific recombinases—have been removed [24]. In addition, key elements in the message might be carried by RNA and proteins that interact with several partners; this is the case of ribosomal RNA and ribosomal proteins in which mutations have an increased risk of disrupting an interaction important for survival. The resulting extreme conservation of rRNA is one reason why it is used for phylogenetic relatedness.

Another apparent difficulty is that natural constraints like the size of the cell or phage might not be accurate enough to be used as reference points. If, however, size constraints lead to regularly spaced natural punctuation marks, the problem becomes one of making these punctuation marks (on which the movies would depend) sufficiently clear. This might be achieved by modifying them, for example, so as to correspond to the exact consensus for a transcriptional activator.

A further difficulty is that *E. nuntius* is likely to be out-competed by its natural competitors which have a billion years of selection on their side. This is not a problem if the conditions do not allow growth and only survival is important. (Note here that bacteria can be resuscitated after millions of years without growth [25].) Being out-competed may not be a problem if *E. nuntius* can occupy fully the niche it is to grow in or if a new niche exists for which it can be designed to fill perfectly. Given the proportion of domestic animals compared to wild animals on Earth, *E. nuntius* could be added to feedstuffs so as to progressively replace the natural gut population.

A variant of our proposal, that we shall only touch on here, would be to use the DNA of a bacterial virus or bacteriophage. The regular nature of the DNA packing into bacteriophage is the object of intense research [26,27,28]. Genes sometimes overlap in bacteriophage [29] so the same sequence can serve more than one function. Multi-functionality decreases the chance of a sequence undergoing radical alteration. This is an important design principle.

## 7. Discussion

Studies of the origin of life on Earth, and the search for it elsewhere, depend on ideas of what cells are. In many cases, these ideas are derived from what microbiologists believed half a century ago. The problem here is that microbiologists' beliefs have changed radically in the last couple of decades [30,31]. Bacteria are now known to be highly structured [32,33,34,35,36] and to live in highly complex communities where they communicate via a variety of mechanisms [37,38]. What is effectively a paradigm-shift has taken the form of a paradigm slide. The new paradigm is beginning to have an effect on origins of life studies [39,40].

A project to construct *E. nuntius* would do more than just create the chance of communicating with other species distant in time and/or space. It would promote creativity and interdisciplinarity in fields as diverse as biology, computer science and astrophysics. For example, by bringing together synthetic biology and cutting-edge microbiology, the project might actually allow origins-of-life studies to make a contribution to these disciplines rather than simply benefit from them. A first step in the construction of a synthetic messenger would be to make matrices out of either the triplet codons or the individual nucleotide bases of a real bacterium and to experiment with the size and number of the frames to see what one gets. In the non-equilibrium approach, one might use the codons of a real bacterium and make frames that vary in both height and width. In the equilibrium approach, one might use four colours for the four nucleotides and construct frames out of a bacterial genome one nucleotide high and a thousand nucleotides wide (because many bacteria are roughly 1000 base pairs in diameter). It is conceivable that even this first step may give something non-random. For example, it is likely that bacteria have been selected so as to obtain optimal compromises between rates and fidelity of translation depending, for example, on growth conditions. One way to achieve this would be if ribosomes could tell the future – which, in principle, they could if a ribosome were to be informed of the codons that it would meet next by the preceding ribosomes (which have already met that codon). Suppose, for example, the tRNA used by ribosome_t_ tells ribosome_t+1_ which amino acid it will need next then, if recently used tRNAs were to increase the affinity of tRNA synthetases for one another, a functioning-dependent hyperstructure might form [32]. Assembly of such a hyperstructure might profit greatly from a non-random distribution of codons in the group of genes that are expressed at any one time.

It is even conceivable that our proposal had already been acted on by an alien species and that the bacteria that made our world actually arrived via panspermia [41] —and contain a message. Reciprocally, absence of a message might be interpreted as indicating that there is no species out there—or back there—that wants to communicate with us. Finally, a still greater ambition would be to send out a computer, or the instructions to build one, rather than a simple message. Such a computer could be based on biology [42], and attempts to build a self-assembling bioputer [43] might have the additional benefit of further enriching the interdisciplinarity at the heart of origin-of-life studies.

## 8. Conclusions

To communicate with alien species, “DNA movies” might be made from the chromosomes of bacteria by representing as a series of frames the distributions of either the individual nucleotides or the codons. Making such movies in what we term the Kilroy project, would require a combination of cutting-edge microbiology and synthetic biology on which the project would draw and to which it would contribute. Indeed, this project might advance significantly our understanding of bacteria and bacterial communities. An irony to which we should be open would be if the vestiges of a message from another species were to exist already in the genomes of cyanobacteria and other bacteria.

## References

[1] Benford G., Bear G. (1995). Old legends. New legends.

[2] Zalasiewicz J. (2009). The Earth After Us.

[3] Palacios M.A., Benito-Pena E., Manesse M., Mazzeo A.D., Lafratta C.N., Whitesides G.M., Walt D.R. (2011). Infobiology by printed arrays of microorganism colonies for timed and on-demand release of messages. Proc. Natl. Acad. Sci. U. S. A..

[4] Bouligand Y., Norris V. (2001). Chromosome separation and segregation in dinoflagellates and bacteria may depend on liquid crystalline states. Biochimie.

[5] Tendulkar A.V., Wangikar P.P. (2011). Characterization and sequence prediction of structural variations in alpha-helix. BMC Bioinformatics.

[6] Kepes F. (2004). Periodic transcriptional organization of the *E. coli* genome. J. Mol. Biol..

[7] Mathelier A., Carbone A. (2010). Chromosomal periodicity and positional networks of genes in *Escherichia coli*. Mol. Syst. Biol..

[8] Cook P.R. (2002). Predicting three-dimensional genome structure from transcriptional activity. Nat. Genet..

[9] Wright M.A., Kharchenko P., Church G.M., Segre D. (2007). Chromosomal periodicity of evolutionarily conserved gene pairs. Proc. Natl. Acad. Sci. U. S. A..

[10] Pedersen A.G., Jensen L.J., Brunak S., Staerfeldt H.-H., Ussery D.W. (2000). A DNA structural atlas for *Escherichia coli*. J. Mol. Biol..

[11] Minsky A. (2004). Information content and complexity in the high-order organization of DNA. Annu. Rev. Biophys. Biomol. Struct..

[12] Baldwin G.S., Brooks N.J., Robson R.E., Wynveen A., Goldar A., Leikin S., Seddon J.M., Kornyshev A.A. (2008). DNA double helices recognize mutual sequence homology in a protein free environment. J. Phys. Chem. B.

[13] Danilowicz C., Lee C.H., Kim K., Hatch K., Coljee V.W., Kleckner N., Prentiss M. (2009). Single molecule detection of direct, homologous, DNA/DNA pairing. Proc. Natl. Acad. Sci. U. S. A..

[14] Wang X., Zhang X., Mao C., Seeman N.C. (2010). Double-stranded DNA homology produces a physical signature. Proc. Natl. Acad. Sci. U. S. A..

[15] Cortini R., Kornyshev A.A., Lee D.J., Leikin S. (2011). Electrostatic braiding and homologous pairing of DNA double helices. Biophys. J..

[16] Inoue S., Sugiyama S., Travers A.A., Ohyama T. (2007). Self-assembly of double-stranded DNA molecules at nanomolar concentrations. Biochemistry.

[17] Randall G.L., Zechiedrich L., Pettitt B.M. (2009). In the absence of writhe, DNA relieves torsional stress with localized, sequence-dependent structural failure to preserve b-form. Nucleic. Acids. Res..

[18] Prentiss M., Kleckner N. (2011). Department of Physic and Department of Molecular and Cellular Biology.

[19] Livolant F.Y., Bouligand Y. (1978). New observations on the twisted arrangement of dinoflagellate chromosomes. Chromosoma.

[20] Chow M.H., Yan K.T., Bennett M.J., Wong J.T. (2010). Liquid crystalline chromosomes: Birefringence and DNA condensation. Eukaryotic Cell.

[21] Chiarabelli C., Vrijbloed J.W., De Lucrezia D., Thomas R.M., Stano P., Polticelli F., Ottone T., Papa E., Luisi P.L. (2006). Investigation of de novo totally random biosequences, part II: On the folding frequency in a totally random library of de novo proteins obtained by phage display. Chem. Biodivers..

[22] Young E., Alper H. (2010). Synthetic biology: Tools to design, build, and optimize cellular processes. J. Biomed. Biotechnol..

[23] Temme K., Zhao D., Voigt C.A. (2010). Refactoring the nitrogen fixation gene cluster with synthetic biology tools.

[24] Posfai G., Plunkett G., Feher T., Frisch D., Keil G.M., Umenhoffer K., Kolisnychenko V., Stahl B., Sharma S.S., de Arruda M. (2006). Emergent properties of reduced-genome *Escherichia coli*. Science.

[25] Fish S.A., Shepherd T.J., McGenity T.J., Grant W.D. (2002). Recovery of 16s ribosomal rna gene fragments from ancient halite. Nature.

[26] Zauberman N., Mutsafi Y., Halevy D.B., Shimoni E., Klein E., Xiao C., Sun S., Minsky A. (2008). Distinct DNA exit and packaging portals in the virus acanthamoeba polyphaga mimivirus. PLoS Biol..

[27] Leforestier A., Livolant F. (2009). Structure of toroidal DNA collapsed inside the phage capsid. Proc. Natl. Acad. Sci. U. S. A..

[28] Leforestier A., Siber A., Livolant F., Podgornik R. (2011). Protein-DNA interactions determine the shapes of DNA toroids condensed in virus capsids. Biophys. J..

[29] Chirico N., Vianelli A., Belshaw R. (2010). Why genes overlap in viruses. Proc. Biol. Sci..

[30] Norris V., Ayala J.A., Begg K., Bouché J.-P., Bouloc P., Boye E., Casaregola S., Cozzone A.J., Crooke E., D'Ari R. (1994). Cell cycle control: Prokaryotic solutions to eukaryotic problems?. J. Theor. Biol..

[31] Bermudes D., Hinkle G., Margulis L. (1994). Do prokaryotes contain microtubules?. Microbiol. Rev..

[32] Norris V., Blaauwen T.D., Doi R.H., Harshey R.M., Janniere L., Jimenez-Sanchez A., Jin D.J., Levin P.A., Mileykovskaya E., Minsky A. (2007). Toward a hyperstructure taxonomy. Annu. Rev. Microbiol..

[33] Defeu Soufo H.J., Reimold C., Linne U., Knust T., Gescher J., Graumann P.L. (2010). Bacterial translation elongation factor EF-Tu interacts and colocalizes with actin-like mreb protein. Proc. Natl. Acad. Sci. U. S. A..

[34] Ptacin J.L., Lee S.F., Garner E.C., Toro E., Eckart M., Comolli L.R., Moerner W.E., Shapiro L. (2010). A spindle-like apparatus guides bacterial chromosome segregation. Nat. Cell Biol..

[35] Llopis P.M., Jackson A.F., Sliusarenko O., Surovtsev I., Heinritz J., Emonet T., Jacobs-Wagner C. (2010). Spatial organization of the flow of genetic information in bacteria. Nature.

[36] Nevo-Dinur K., Nussbaum-Shochat A., Ben-Yehuda S., Amster-Choder O. (2011). Translation-independent localization of mRNA in *E. coli*. Science.

[37] Shapiro J.A. (2007). Bacteria are small but not stupid: Cognition, natural genetic engineering and socio-bacteriology. Stud. Hist. Philos. Biol. Biomed. Sci..

[38] Dubey G.P., Ben-Yehuda S. (2011). Intercellular nanotubes mediate bacterial communication. Cell.

[39] Hunding A., Kepes F., Lancet D., Minsky A., Norris V., Raine D., Sriram K., Root-Bernstein R. (2006). Compositional complementarity and prebiotic ecology in the origin of life. Bioessays.

[40] Norris V., Root-Bernstein R. (2009). The eukaryotic cell originated in the integration and redistribution of hyperstructures from communities of prokaryotic cells based on molecular complementarity. Int. J. Mol. Sci..

[41] Wickramasinghe C. (2004). The universe: A cryogenic habitat for microbial life. Cryobiology.

[42] Tamsir A., Tabor J.J., Voigt C.A. (2011). Robust multicellular computing using genetically encoded nor gates and chemical ‘wires’. Nature.

[43] Norris V., Zemirline A., Amar P., Audinot J.N., Ballet P., Ben-Jacob E., Bernot G., Beslon G., Cabin A., Fanchon E. (2011). Computing with bacterial constituents, cells and populations: From bioputing to bactoputing. Theory Biosci..

